# Editorial: Ethical and regulatory challenges in genetic and genomic research involving stored biological specimens

**DOI:** 10.3389/fgene.2022.1062188

**Published:** 2022-10-21

**Authors:** Nut Koonrungsesomboon, Kenji Hirayama

**Affiliations:** ^1^ Department of Pharmacology, Faculty of Medicine, Chiang Mai University, Chiang Mai, Thailand; ^2^ Clinical Research Center for Food and Herbal Product Trials and Development (CR-FAH), Faculty of Medicine, Chiang Mai University, Chiang Mai, Thailand; ^3^ Department of Immunogenetics, Institute of Tropical Medicine, Nagasaki University, Nagasaki, Japan

**Keywords:** ethics, biobank, genetics, genomics, regulations, biospecimen, biological specimens

Genetic/genomic research requires access to large quantities of biological specimens and related data, so biobanks are of paramount importance for the advancement of such research and precision medicine. However, biobank research and genetic/genomic research have several unique features that raise certain ethical and regulatory challenges, many of which have been much debated in an effort to find proper solutions applicable to international and local settings. Respect for persons, privacy, and confidentiality are among the most important ethical and regulatory issues in this regard. It is, therefore, imperative that ethical and regulatory frameworks be in line with and able to facilitate the development of biobanks and the conduct of genetic/genomic research.

The success of biobanks and genetic/genomic research on human biological specimens depends largely on the social perception of human tissues and cultural beliefs about the body, and the willingness to donate human biological specimens for research purposes. Majchrowska et al. undertook a quantitative study to distinguish different sociocultural categories of human tissues and the characteristics of potential donors based on their willingness to donate their biological specimens for research. The authors found that the willingness to donate biological specimens for research is largely shaped by the sociocultural perception of certain parts of the body and tissues. The lower the sense of personal connection to a given sort of tissue or part of the body, the more likely one is to donate such biological specimens for research purposes. Another key factor contributing to the willingness to donate human biological specimens for research is the social trust in researchers and research governance. The work from Majchrowska et al. is useful for stakeholders looking to improve the effectiveness of cooperation between biobanks/researchers and potential donors, as well as to establish an effective approach for the collection of biological specimens.

Since public trust is crucial for the development of biobanks, data privacy and security are of the utmost importance in this regard. The General Data Protection Regulation (GDPR) is a pertinent piece of legislation that harmonizes data privacy laws across the European Union (EU), including organizations/enterprises that collect data on EU citizens. The GDPR allows for the development of codes of conduct for processing personal data. Krekora-Zając et al. share their experiences with a given scenario in Poland and provide recommendations for creating codes of conduct for processing personal data in biobanks. In brief, code developers should determine the code’s purpose and application scope, while considering various guidelines on research ethics. The minimum technical regulations for safety management in data processing should be determined. Then, multi-stage public consultations should be carried out during the process of code development to seek input from broad stakeholders. After all, the code of conduct ought to have a clear layout and understandable language, with sample explanations that make it applicable to real-world practice. Based on these recommendations, biobanks shall uniformly handle data, make data transfers more appropriate, and ensure that the rights of the donors to whom the data pertain are respected. From a long-term perspective, the code of conduct for data processing shall promote public trust in biobanks and genetic/genomic research involving stored biological specimens and related data.

The GDPR also emphasizes the importance of appropriate safeguards when personal data may be processed for research purposes. However, guidance on what appropriate safeguards could be is limited within the GDPR. Staunton et al., thus, examined relevant ethical guidelines and regulations regarding appropriate safeguards in the context of biobanks and genetic/genomic research involving biological specimens and related data. Based on their analysis, the authors propose six possible safeguards, each of which is dependent on specific research and its related context. First, informed consent should be appropriately designed to cover the context of collecting and using biological specimens and related data. Second, independent review and oversight are required for the establishment of a biobank/databank, as well as for any research involving stored biological specimens and related data. Third, accountable processes should be in place to govern access and use of stored biological specimens and related data for research. Fourth, policies on all aspects of the collection and use of stored biological specimens and related data should be clear and transparent. Fifth, security measures (e.g., coding and encryption) should be set up to prevent unauthorized access to stored biological specimens and related data. Sixth, education and training for those who handle biological specimens and related data are necessary and should be ongoing. The proposal from Staunton et al. offers an integrated bioethics approach to data protection in biobanks and genetic/genomic research involving stored biological specimens and related data.

Last but not least, certain advanced therapy medicinal products (e.g., monoclonal antibodies produced from hybridoma technologies) sometimes raise legal conflicts in the trajectory of patent claims and commercial interests of such products. The ethical debate on the fairness of such interests and the disclosure of relevant information to donors is still under scrutiny. The donors may not have a chance to make their choice regarding commercial usage of their biological specimens unless information about whether their biological specimens may be used for commercial profit and whether they shall or shall not share in this commercial profit is adequately disclosed. D’Abramo et al. describe concerns regarding biomolecular prospecting and informative gaps, based on fieldwork research together with an epistemological, historical, and ethical analysis of informed consent for clinical trials for monoclonal antibodies and biobank research. The authors make several noteworthy remarks. For example, the historical analysis reveals that the patent regime governing monoclonal antibodies is prone to the practice of withholding commercial-related information without the donor’s consent. The findings of the fieldwork provide evidence in favor of this; for instance, informed consent documents are often complicated with few explanations about technologies and infrastructures underpinning clinical trials and biobank research.

In sum, this Research Topic provides a collection of high-quality research papers describing ethical and regulatory challenges in genetic/genomic research involving stored biological specimens and related data. We anticipate that this compilation will aid the scholarly community in better addressing these ethical and regulatory concerns.

